# Description of plant tRNA-derived RNA fragments (tRFs) associated with argonaute and identification of their putative targets

**DOI:** 10.1186/1745-6150-8-6

**Published:** 2013-02-12

**Authors:** Guilherme Loss-Morais, Peter M Waterhouse, Rogerio Margis

**Affiliations:** 1 Universidade Federal do Rio Grande do Sul, Centro de Biotecnologia, Predio 43431, Sala 213, POBox 15005, Porto Alegre, RS, Brazil; 2 The University of Sydney, Sydney, NSW, Australia

**Keywords:** tRNAs, Small RNA, tRFs, tRNA-derived RNA fragments, Argonaute and Arabidopsis

## Abstract

tRNA-derived RNA fragments (tRFs) are 19mer small RNAs that associate with Argonaute (AGO) proteins in humans. However, in plants, it is unknown if tRFs bind with AGO proteins. Here, using public deep sequencing libraries of immunoprecipitated Argonaute proteins (AGO-IP) and bioinformatics approaches, we identified the *Arabidopsis thaliana* AGO-IP tRFs. Moreover, using three degradome deep sequencing libraries, we identified four putative tRF targets. The expression pattern of tRFs, based on deep sequencing data, was also analyzed under abiotic and biotic stresses. The results obtained here represent a useful starting point for future studies on tRFs in plants.

## Findings

Small RNAs are usually ~20 nucleotides long. Regardless of their genomic origin, small RNAs can regulate gene expression by acting as siRNAs to direct DNA methylation [1] or by acting as microRNAs to direct post transcriptional gene silencing (PTGS) [2]. microRNAs are the most studied class of small RNAs [3]. Moreover, the key enzymes related to small RNA biogenesis, such as Dicer-Like (DCL) and AGO proteins, and their roles in PTGS have been well described [2].

The recent development of high-throughput sequencing technology has improved the identification of other types of small RNAs [4], like tRNA-derived RNA fragments (tRFs) [3]. The proposed nomenclature of tRFs is based on the regions of tRNA cleavage, including 3' U tRFs that are processed from pre-tRNAs and consist of the sequence between the cleavage site and the RNA PolIII run-off poly(U) tract [5]. Mature tRNA can generate two main types of tRFs: one processed from the 5' end (5' tRFs) and another from the 3' end, harboring the added CCA sequence (3' CCA tRFs) [5].

The tRFs were first discovered in cultured *Hela* cells [6]. Subsequent work in other animal tissues showed that tRF biogenesis may involve RNAse Z [5] as well as Dicer processing [6-8].

Recently, it has been suggested that there might be cross-talk between tRFs and the canonical small RNA pathway, which includes the microRNAs [5]. Another exciting finding was that of the association of tRFs with AGO proteins [6,7] and the demonstration of a RNAi-type trans-silencing induced by a 3' CCA tRF using a reporter gene [7].

At present, only three works show the existence of tRFs in plants. In *Arabidopsis thaliana,* the 5' tRF of AspGTC and the 5' and 3' CCA tRFs of GlyTCC tRNAs were found to be overexpressed in root tissues treated with phosphate deprivation [9]. In rice, the 5' AlaAGC and ProCGG tRFs demonstrated differential expression in the callus and leaves [4]; in barley, the HisGTG tRF was the most abundant of all the small RNAs [10]. However, the possible association of tRFs to AGO proteins and their potential contribution to the RNAi pathway were not analyzed in either of the previous studies.

**Figure 1:**
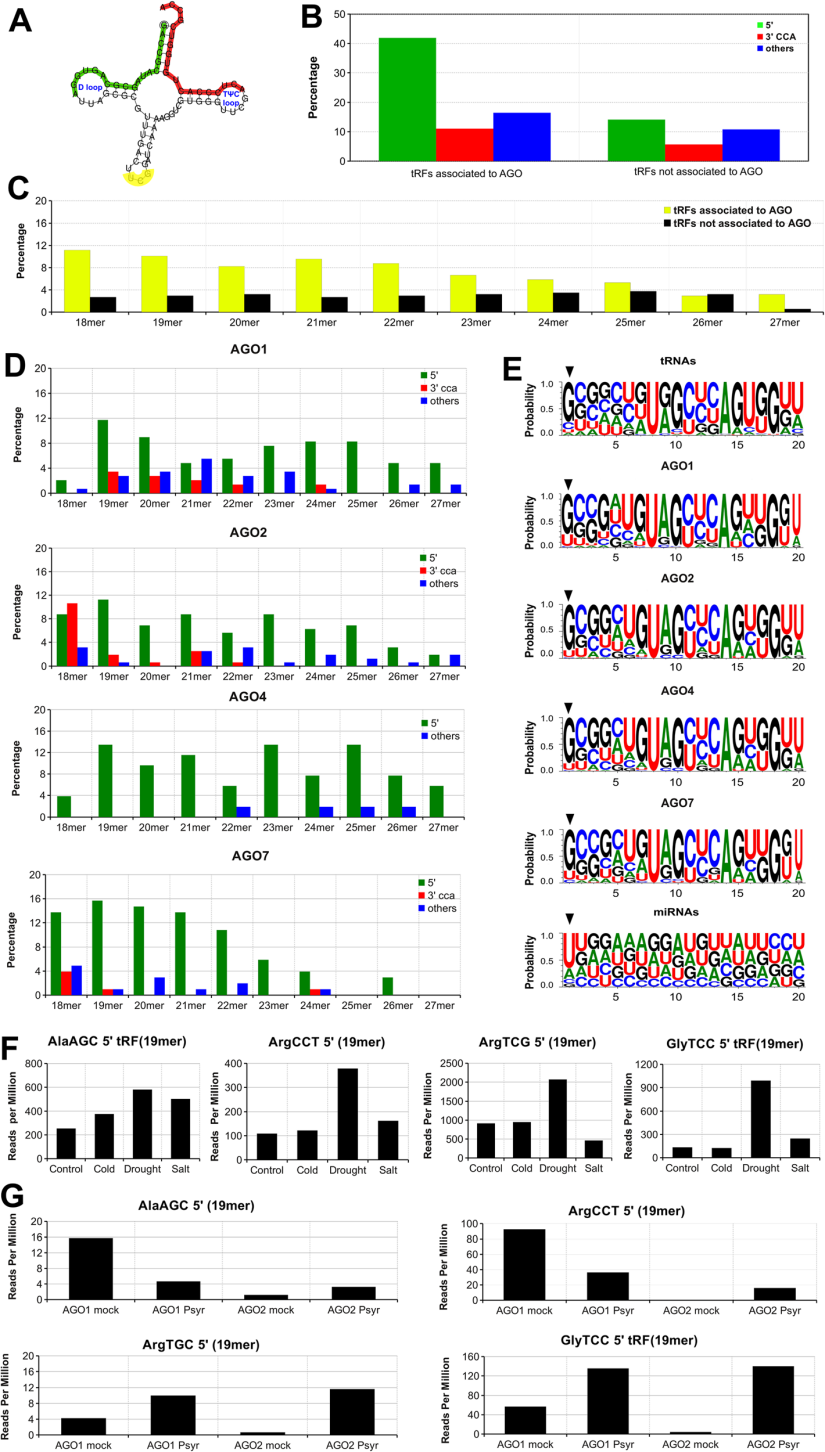
**tRNA-derived RNA fragments (tRFs) from *Arabidopsis thaliana *associated with AGO. A) **Schematic representation of ArgTCG tRNA showing the 5' tRF in green, the 3' CCA tRF in red and the anti-codon in yellow. The D and TΨC loops are also shown. **B)** tRF class diversity of AGO-associated tRFs and unassociated ones. **C)** Length diversity of AGO-associated tRFs and unassociated ones. **D)** tRF length diversity of AGO1, 2, 4 and 7 IP deep sequencing libraries. **E)** Logo representation of the first 20 nucleotides of tRNAs, AGO1-IP tRFs, AGO2-IP tRFs, AGO4-IP tRFs, AGO7-IP tRFs and the *A. thaliana* microRNAs (miRBase v. 18). The black arrowheads indicate the first nucleotide at the 5' end. **F)** Expression pattern of AlaAGC, ArgCCT, ArgTCG and GlyTCC 5' tRFs in control (untreated), drought (40-50% relative water content), cold (5°C for 24 hours), and salt (200 mM of NaCl for 5 hours) conditions. The expression patterns are shown in reads per million, where the tRF frequency was divided by the total number of reads and multiplied by one million. **G)** Expression pattern of AlaAGC, ArgCCT, ArgTCG and GlyTCC 5' tRFs in biotic stress. The expression patterns are also shown in reads per million. The leaves were inoculated with mock solution (10 mM MgCl_2_) or *Pseudomonas syringae* (2 x 107 cfu/ml). The inoculated leaves were collected 14 hours after inoculation.

The work described here was designed to identify putative AGO-associated tRFs in *Arabidopsis thaliana* by analyzing public small RNA deep sequencing libraries, including those from AGO immunoprecipitation (AGO-IP) assays. Putative tRF target sequences were also found by examining *Arabidopsis* public degradome sequencing libraries. The expression patterns of tRFs under abiotic and biotic stresses were also analyzed. The present work focused on 5' and 3' CCA tRFs in *A. thaliana*, but sequences derived from the central regions of the tRNA were also searched (see methods) (Figure 1A).

We inspected AGO1, 2, 4, 6, 7 and 9 IP libraries [See Additional file 1: Table S1] and found tRFs in the AGO1, 2, 4 and 7 IP libraries (Figure 1B,-D) [See Additional file 2: Table S2]. Both, 5' and 3' CCA *Arabidopsis* tRFs were associated with AGO, mirroring previous results in mammalian systems [6,7]. Interestingly, tRFs from the central part of the tRNA were also detected (Figure 1B,-D), although 5' tRFs formed the most abundant class [4,6,9] and showed the highest sequence diversity (Figure 1B,-D).

Examining the AGO-associated and unassociated tRFs (Figure 1C) [See Additional file 3: Figure S1] revealed a bias in size distribution, with the AGO-associated tRFs being predominantly 18-22 (nt) in length (Figure 1C) and the AGO-associated 5’tRFs being predominantly 19 mers (Figure 1D) [See Additional file 3: Figure S1]. This is very similar to the situation in *Hela* cells [6].

The predominant 5' terminal nucleotide of microRNAs is a uracil [11], and this first base is thought to be a major determinant for loading onto AGO1. AGO2 and AGO4 preferentially recruit small RNAs with a 5' terminal A [12,13]. However, the most common 5' nucleotide of 5' tRFs is G (Figure 1E). Takeda et al. (2008) suggested that *Arabidopsis* may have an AGO gene with a preference for microRNAs starting with guanine [12]; however, it does not seem to be applicable to tRFs.

Further, to investigate if the 5' tRFs associated with AGOs act in the RNAi pathway in plants, as has been suggested in animals [7], we looked for tRF targets in *Arabidopsis* using a well-known plant microRNA target prediction tool coupled with degradome analyses. This analysis identified four possible target genes [See Additional file 4: Table S3]. However, this method assumes that the mechanism and characteristics of tRF target recognition are similar to those for microRNAs, which remains to be demonstrated. Indeed, it is possible that tRFs may play a role in DNA and chromatin modification because we found that tRFs associated with AGO4 (Figure 1D), which is known to be involved in this process [12].

In order to inspect the expression pattern of tRFs in abiotic stress treatments, we conducted an analysis of the AlaAGC, ArgCCT, ArgTCG and GlyTCC 5' tRFs, using the available deep sequencing data (Figure 1F). Drought conditions enhanced the expression of the four tRFs, including the GlyTCC 5' tRF, which is already known to be up-regulated in response to phosphate deprivation [9]. Hsieh et al. (2009) discussed that tRFs accumulate in a developmentally regulated manner and become dominant in specific tissues or under specific stress conditions [9]. Thus, the 5' GlyTCC seems to be dominant in both phosphate deprivation and drought treatment.

The expression pattern of tRFs under biotic stress in plants is currently unknown. In order to identify tRFs that respond to biotic stress, we conducted an expression analysis of the same four 5' tRFs in AGO1 and AGO2 immunoprecipitated deep sequencing libraries from *Arabidopsis* infected with *Pseudomonas syringae* or mock solution (Figure 1G). The four 5' tRFs showed increased expression in infected AGO2-IP libraries (Figure 1G). AGO2 is a protein of unknown function [2]; however, this protein was recently characterized as being strongly induced by *P. syringae* infection [14]. This work also investigated the microRNA pathway and showed that the expression levels of miR393*, which associated with AGO2-IP and targets a transcript related to exocytosis, was enhanced in *P. syringae* infection assay [14]. Here, we found an increase in expression of 5' tRFs in the AGO2-IP, indicating a possible role for 5' tRFs in *P. syringae* infection. However, more experiments should be performed.

## Conclusions

Small RNAs are important regulators of gene expression, and recent advances in sequencing and bioinformatics techniques have stimulated the discovery of new classes of small RNAs. Here, we report for the first time that tRNA-derived RNA fragments (tRFs) associate with AGO proteins in plants. The first nucleotide does not seem to determine which 5' tRF is directed to which AGO protein, as observed in microRNAs. However, there is some enrichment of uridine at the 5' end. Moreover, we identified putative tRF targets and analyzed the expression of tRFs under abiotic and biotic stresses. The results presented in this study can be considered as valuable support for future studies on the complex networks involved in tRF-mediated gene regulation in plants.

## Methods

In order to find tRFs associated with AGO, 34 deep sequencing libraries were retrieved from the GEO database (http://www.ncbi.nlm.nih.gov/geo/) [15], including 25 libraries of AGO-IP and three degradome libraries [See Additional file 1: Table S1]. We identified a third tRF class, corresponding to tRFs originating from the internal sequences of the tRNA. These reads did not map to the very first nucleotide of 5' tRFs or the very last nucleotide of 3' CCA tRFs.

The bioinformatics approaches used to identify tRFs associated with AGO were shown in Additional file 5: Figure S2. Briefly, reads from a control (GSM647184) library were mapped against all mature *Arabidopsis* tRNAs previously obtained from the TAIR database (http://www.arabidopsis.org), resulting in putative tRFs. Further, the putative tRFs were used as a query to inspect the AGO-IP libraries. The putative tRFs, which were found in the AGO-IP and had a frequency of more than 10 reads, were retrieved and considered AGO-associated tRFs. Later, the AGO-associated tRFs were used for target prediction against all *Arabidopsis* transcripts using the psRNATarget tool (http://plantgrn.noble.org/psRNATarget/). The degradome libraries were used to confirm possible target cleavage, lowering the false positive rate in the tRF target prediction.

## Competing interests

The authors declare that they have no competing interests.

## Authors’ contribution

GLM conceived the idea, performed the computational work and wrote the paper. PMW and RM contributed to the interpretation of the results and the preparation of manuscript. All authors read and approved the final manuscript.

## Authors’ response

The main criticism made by both referees concerned the necessity of experimental validation of predicted targets of Arabidopsis tRFs in order to demonstrate the reliability of the predictions made.

As stated, the target prediction was performed using psRNAtarget. The putative hibrydization site between each tRF and its corresponding target transcript were searched in public Arabidopsis DEGRADOME sequencing libraries. The authors consider that the presence of the corresponding sequences in the DEGRADOME libraries provides reliability, in a first instance, to the *in silico* predicted targets. Authors agree that RACE and other experiments would be required to assure the exactitude and extent of tRFs targets, but also consider that these experiments would be out of the scope of the present work. It is important to remark that along the reviewing process of this work, a third paper was published about the identification of tRFs in plants, but without any comments about their association to AGO proteins. This work was incorporated in our list of references.

## Reviewer number: 1

Report form:

The authors screened existing Arabidopsis databases of the small RNAs associated with AGO to find tRNA-derived small RNAs (tRFs) and to identify their potential targets. The work is rather modest in scope and would greatly benefit from experimental validation of the tRF targets. The outcome of the work could be useful for those studying RNAi pathways in plants.

## Reviewer number: 2

Report form:

This is the first report of tRNA fragments associated with Argonaute in plants and accordingly of interest. The manuscript would be improved if the authors were explicit about the reliability of the tRF target prediction.

## Supplementary Material

Additional file 1: Table S1 Details of the deep sequencing libraries used in the present analyses. Click here for file

Additional file 2: Table S2 List and details of the tRFs identified in the present work. Click here for file

Additional file 3: Figure S1 Raw read frequencies of AGO1, 2, 4 and 7 immunoprecipitated libraries. Raw frequency of the tRFs is also shown. The most expressed reads or tRFs of each AGO-IP library are underlined. Click here for file

Additional file 4: Table S3 Report the predicted tRFs targets validated by degradome analyses. Click here for file

Additional file 5: Figure S2 Fluxogram showing the bioinformatics approaches for identification and tRF target prediction of AGO-associated tRFs. The putative targets were used as a reference to screen degradome libraries. The degradome reads, which were mapped to the approximate central portion of the tRF target recognition site and show at least one match and one wobble in tRF:target pairing, were retrieved. So far, putative targets were validated by degradome analyses. Click here for file
