# Supplementary figures and images for: Description of plant tRNA-derived RNA fragments (tRFs) associated with argonaute and identification of their putative targets
Source: Biol Direct. 2013 Feb 12;8:6. doi: 10.1186/1745-6150-8-6 (PMC3574835; doi:10.1186/1745-6150-8-6)

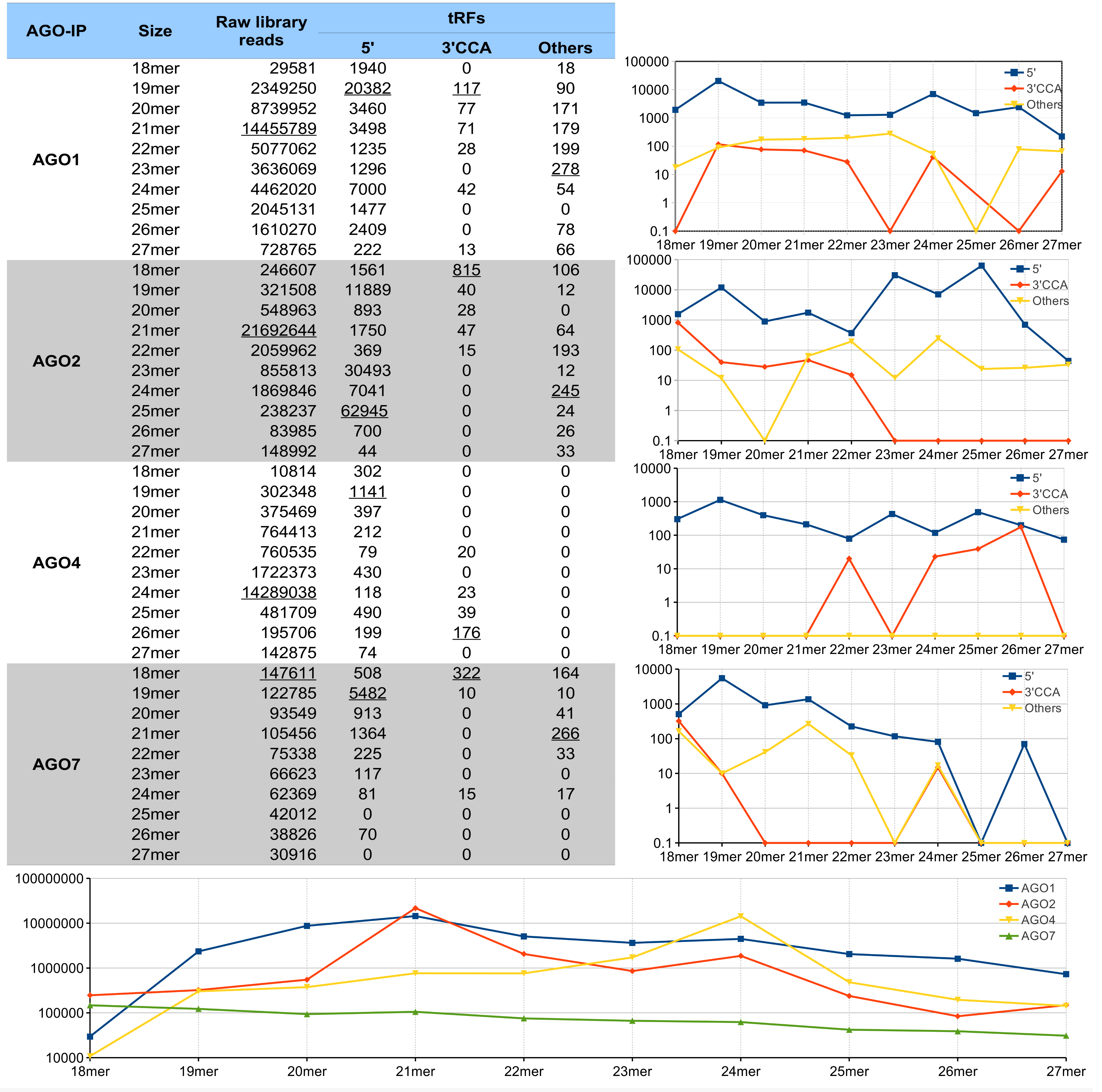

Supplement: Additional file 3: Figure S1 — Raw read frequencies of AGO1, 2, 4 and 7 immunoprecipitated libraries. Raw frequency of the tRFs is also shown. The most expressed reads or tRFs of each AGO-IP library are underlined. [file 1745-6150-8-6-S3.tiff]

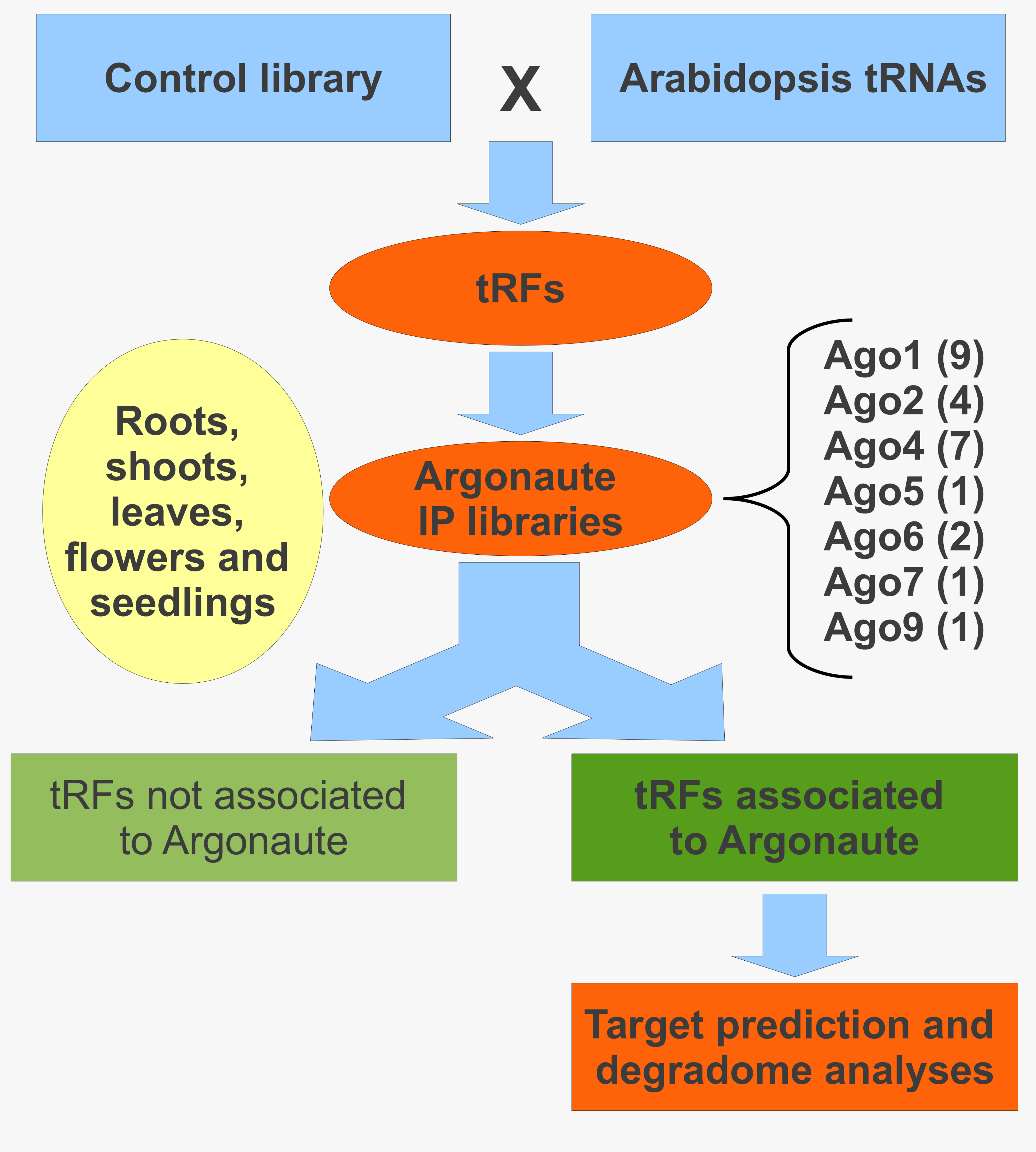

Supplement: Additional file 5: Figure S2 — Fluxogram showing the bioinformatics approaches for identification and tRF target prediction of AGO-associated tRFs. The putative targets were used as a reference to screen degradome libraries. The degradome reads, which were mapped to the approximate central portion of the tRF target recognition site and show at least one match and one wobble in tRF:target pairing, were retrieved. So far, putative targets were validated by degradome analyses. [file 1745-6150-8-6-S5.tiff]
