# Supplementary material for: Description of plant tRNA-derived RNA fragments (tRFs) associated with argonaute and identification of their putative targets
Source: Biol Direct. 2013 Feb 12;8:6. doi: 10.1186/1745-6150-8-6 (PMC3574835; doi:10.1186/1745-6150-8-6)
Supplement: Additional file 4: Table S3 — Report the predicted tRFs targets validated by degradome analyses. [file 1745-6150-8-6-S4.pdf]

Additional File 4: Degradome analyses of predicted tRFs targets. The tRFs acronyms, target loci, tRFs:target alignments, target descriptions, GO biological processes and GO molecular functions. The region of predicted target cleavage is showed by underlined regions.

tRFs	Target loci	tRFs:target alignments	Target Descriptions	GO Biological Processes	GO Molecular Functions
AlaAGC (19mer)	AT3G61060.1	tRF 17 UAGACUCGAUGUAGGGG 1<br>:::::.:::.:::.:.:<br>Target 1206 AUCUGGGCUGCAUUCUC 1222	Phloem protein 2-A13 (PP2-A13)	Involved in response to wounding	Functions in carbohydrate binding
ArgCCT (19mer)	AT3G05050.1	tRF 19 AGGUGACUCGAUGUCCGCG 1<br>:.:::::::: :::::.<br>Target 1164 UUCACUGAGCAACAGGUUA 1182	Protein kinase superfamily protein	Involved in protein phosphorylation	Funtions in serine/threonine kinase activity
ArgTCG (19mer)	AT2G24790.1	tRF 18 GGUGACGCGAUACGCCAG 1<br>::.:::::.::::::<br>Target 261 CCGCUGCGUUAUGCGUCA 278	COL3 (CONSTANS-LIKE 3) transcription factor	Involved in regulation of photomorphogenesis, regulation of transcription	Functions in protein binding, zinc ion binding
GlyTCC (19mer)	AT3G57280.1	tRF 18 GGCAACCUGAUGUCUGCG 1<br>:.::::::.::: :..:.<br>Target 925 CUGUUGGAUUACUGGUGU 942	Transmembrane protein 14C	unknown	unknown
